# Enhanced In Vivo Antinociceptive Activity and GC–MS Chemical Profiling of In Vitro-Grown *Acmella radicans* Plants Elicited with *Rhizopus microsporus*

**DOI:** 10.3390/molecules31162906

**Published:** 2026-08-20

**Authors:** Giovanna Hernández-Aubert, Rosa Mariana Montiel-Ruiz, Israel Hurtado-Díaz, José Antonio Silva-Guzmán, José Guillermo Torres-Rendón, Ricardo Manríquez-González, Francisco Cruz-Sosa, Antonio Bernabé-Antonio

**Affiliations:** 1 Department of Wood, Pulp and Paper, University Center of Exact Sciences and Engineering, University of Guadalajara, Km 15.5 Guadalajara-Nogales, Col. Las Agujas, Zapopan 45100, Jalisco, Mexico; lizette.haubert@alumnos.udg.mx (G.H.-A.); jose.torres@academicos.udg.mx (J.G.T.-R.); ricardo.manriquez@academicos.udg.mx (R.M.-G.); 2 South Biomedical Research Center, Mexican Institute of Social Security (IMSS), Xochitepec 62790, Morelos, Mexico; rosa.montiel@imss.gob.mx; 3 Department of Molecular Biology and Genomics, University Center for Health Sciences, University of Guadalajara, Sierra Mojada 950, Col. Independencia Oriente, Guadalajara 44340, Jalisco, Mexico; 4 Department of Biotechnology, Metropolitan Autonomous University-Iztapalapa Campus, Av. Ferrocarril de San Rafael Atlixco 186, Col. Leyes de Reforma 1ª. Sección, Alcaldía Iztapalapa, Mexico City 09310, Mexico; cuhp@xanum.uam.mx

**Keywords:** alkamides, biotic elicitation, formalin test, lipophilic fractions

## Abstract

Pain remains a major global health challenge, driving the search for safer analgesics from medicinal plants. Although *Acmella radicans* is traditionally used for pain relief, the effects of fungal elicitation on its chemical profile and antinociceptive activity remain unknown. Therefore, this study evaluated the effects of *Rhizopus microsporus* elicitation on the chemical profile and in vivo antinociceptive activity of lipophilic fractions from in vitro-grown *A. radicans*. In vitro-grown plants were biotically elicited with aqueous mycelial extracts of *R. microsporus*. Lipophilic fractions obtained from ethanolic extracts were analyzed by GC–MS and evaluated for antinociceptive activity using the mouse formalin test. Elicitation with a 0.5% mycelial extract (48 h fungal culture, 48 h exposure) increased ethanol extract yield to 18.8%, compared with 15.3% in non-elicited in vitro plants and 6.1% in wild plants. GC–MS analysis tentatively identified 62 metabolites and indicated marked changes in the lipophilic chemical profile, including lower relative abundance of spilanthol and higher relative abundances of phenethyl alkamides, stigmasterol, *β*-sitosterol, and 2,4-di-tert-butylphenol, along with the tentative identification of (2*E*,4*E*)-*N*-(2-phenylethyl)deca-2,4-dienamide, a compound not previously reported in the genus *Acmella*. LFEP showed the highest efficacy among the plant fractions, reducing neurogenic pain by 60.3% versus 88.7% with tramadol, while its significant effect in the inflammatory phase approached that of diclofenac (54.1% inhibition), with tramadol producing the strongest inhibition (81.0%). Fungal elicitation modified the lipophilic chemical profile of *A. radicans* and enhanced its in vivo antinociceptive activity, supporting its application as a biotechnological strategy to improve the pharmacological potential of medicinal plant in vitro cultures.

## 1. Introduction

Pain represents a major global public health challenge, affecting approximately one in five people worldwide. Defined as an unpleasant sensory and emotional experience associated with actual or potential tissue damage, pain serves as a protective mechanism that alerts the body to potentially harmful stimuli [1]. Beyond its clinical consequences, pain reduces physical and functional capacity, increases work absenteeism, diminishes quality of life, and imposes a substantial socioeconomic burden [2,3,4]. In this context, there is growing interest in the development of safer and more effective analgesic therapies, as conventional analgesics, including nonsteroidal anti-inflammatory drugs (NSAIDs) and opioids, present important limitations. NSAIDs may cause severe gastrointestinal and renal adverse effects [5,6], whereas opioids are associated with dependence and respiratory depression [7,8].

Among medicinal plants, species belonging to the genus *Acmella* (Asteraceae) represent a promising source of bioactive metabolites. This genus comprises more than 60 species distributed throughout Mexico and Central America and is characterized by the production of aliphatic alkamides, compounds recognized for their anti-inflammatory, anesthetic, and antinociceptive activities [9,10]. Among them, *Acmella radicans* (Jacq.) R.K. Jansen, commonly known in Mexico as toothache herb, has traditionally been used to relieve toothache and sore throat [11]. The analgesic properties attributed to this species have been mainly associated with the presence of alkamides, particularly spilanthol, which inhibits cyclooxygenase-2 (COX-2), modulates TRPV1 channels, and exhibits affinity for cannabinoid CB1 and CB2 receptors [12,13,14]. Furthermore, GC–MS studies have identified some alkamides in *A. radicans*, supporting its potential as a source of bioactive compounds [12].

To reduce the pressure on wild populations of medicinal plants [15], in vitro culture has emerged as a biotechnological alternative that enables the production of plant biomass under controlled, season-independent, and aseptic conditions [16]. Among the strategies used to enhance the accumulation of specialized metabolites, biotic elicitation has proven effective by activating signaling pathways and genes involved in defense-related biosynthetic processes through microbe-derived signaling [17,18]. Mycelial extracts of *Rhizopus oligosporus* have been reported to markedly enhance the accumulation of capsaicin and related capsaicinoids, a group of specialized alkamides, in *Capsicum frutescens* cell suspension cultures [19]. More broadly, fungal elicitors have been shown to stimulate the production of specialized metabolites in plants through the activation of defense-related metabolic pathways [20,21]. Notably, *R. microsporus* has been shown to induce the accumulation of defense-related diterpenoid phytoalexins, including kauralexins, in maize [20,22].

However, to the best of our knowledge, no studies have evaluated the effects of *R. microsporus* elicitation on the chemical profile and antinociceptive activity of in vitro-grown *A. radicans*. Therefore, the present study evaluated the effects of biotic elicitation with *R. microsporus* on the chemical profile and in vivo antinociceptive activity of lipophilic fractions obtained from in vitro-grown *A. radicans*, compared with wild plants and reference analgesic drugs, as a sustainable biotechnological approach to enhance the pharmacological potential of this medicinal species.

## 2. Results and Discussion

### 2.1. Growth Kinetics and TLC Chemical Profile of R. microsporus

**Figure 1:**
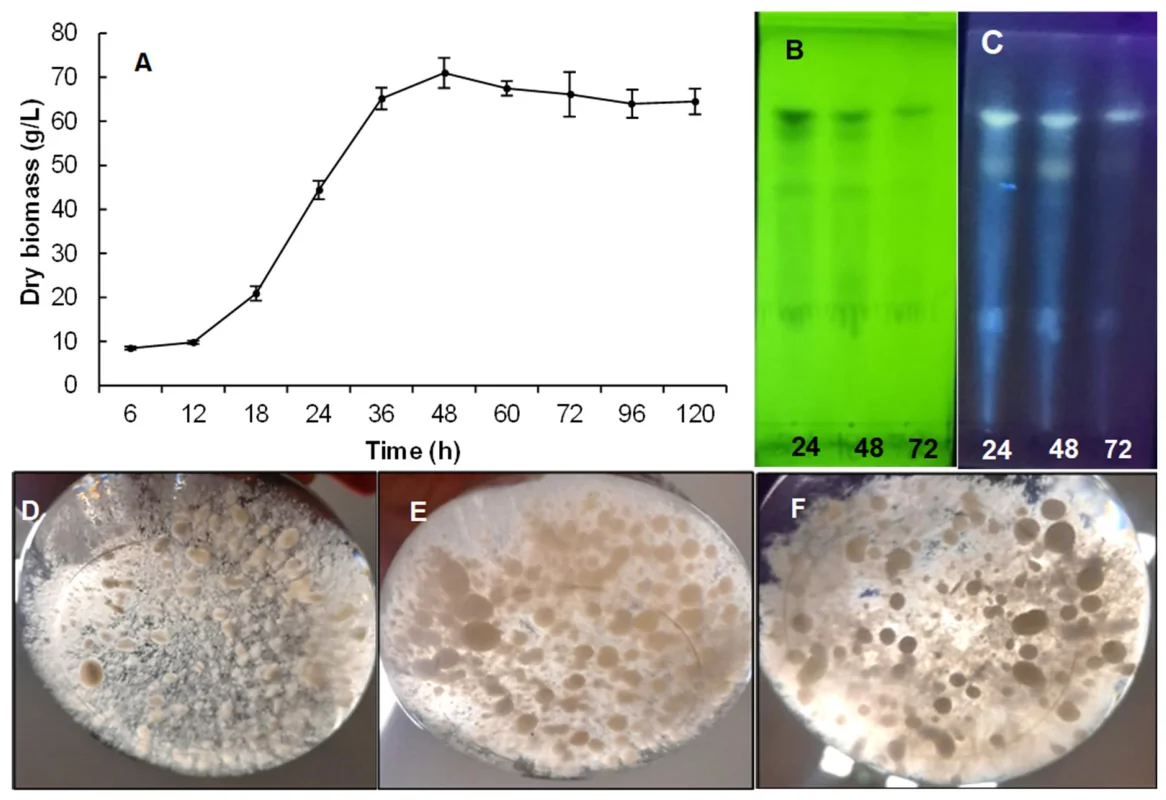
Characterization of *Rhizopus microsporus* during its growth. (**A**) Growth kinetics in potato dextrose broth (PDB). Values represent the mean ± SD of three independent experiments. (**B**,**C**) TLC profiles of aqueous extracts from mycelial biomass harvested at 24, 48, and 72 h. Silica gel 60 F_254_; 100% methanol as the mobile phase; visualization under UV light at 254 nm (**B**) and 365 nm (**C**). (**D**–**F**) Appearance of *R. microsporus* cultures at 24 h (exponential phase), 48 h (early stationary phase), and 72 h (late stationary phase), respectively.

The growth and characterization of *R. microsporus* are shown in Figure 1A–F, which exhibited the characteristic sigmoidal growth pattern of filamentous fungi in potato dextrose broth under submerged culture conditions. Following a brief adaptation phase, the fungus entered the exponential phase at 12 h, reached maximum biomass during the early stationary phase at 48 h, and showed an initial decline after 60 h (Figure 1A).

The decline observed after the stationary phase may be attributed to nutrient depletion and the accumulation of inhibitory metabolites, as commonly reported for filamentous fungi [23]. Mycelial biomass became visible from 6 h onward, while pellet formation was first observed at 24 h; the pellets reached 3–8 mm in diameter and developed a homogeneous, porous morphology (Figure 1D–F), which has been reported to improve nutrient and oxygen diffusion and provide favorable conditions to produce bioactive fungal metabolites [24]. TLC analysis of the mycelial extracts (Figure 1B,C) revealed fluorescent bands, suggesting the presence of fluorophoric compounds, possibly flavins or quinones, as well as medium- to high-polarity metabolites potentially involved in fungal stress signaling [25]. Fluorescence intensity under 365 nm was greatest in the 24 and 48 h extracts, indicating a higher accumulation of these metabolites during the exponential and early stationary phases. However, TLC performed using 100% methanol as the mobile phase is primarily suitable for detecting relatively polar, low-molecular-weight compounds and does not allow the identification of specific elicitor-active components, particularly high-molecular-weight fungal elicitors such as *β*-glucans, chito-oligosaccharides, elicitor proteins, and cell-wall polysaccharides. These components have been reported in *Rhizopus* spp. and are widely recognized as elicitors of plant defense responses [26,27]. Therefore, although differences were observed among the chemical profiles of the aqueous mycelial extracts, the metabolic response of *A. radicans* cannot be attributed to individual fungal signal molecules. Further characterization of the mycelial extract is required to identify the components involved in the elicitation response.

### 2.2. Plant Growth and Biomass Yield

*A. radicans* plants grown on gelled MS medium developed slender, erect stems, small bright-green leaves, and a compact growth habit, displaying the characteristic morphology of the species. After five days in liquid medium, the plants exhibited firmer leaves, thicker stems, and a 71% increase in aerial dry biomass (120 mg/plant) compared with plants grown on gelled medium (70 mg/plant). This increase is likely attributable to greater nutrient availability, improved oxygen transfer, and the absence of the physical constraints imposed by gelled media, conditions that promote plant growth, biomass accumulation, and secondary metabolism [15,28].

### 2.3. Yield of Extracts and Fractions

The ethanolic extract obtained from plants grown on gelled MS medium (PGE) showed a yield of 12.9%, exceeding the 6.1% previously reported for wild *A. radicans* plants [12]. The yield further increased to 15.3% in the ethanolic extract from plants grown in liquid medium (NEE), consistent with the greater biomass production observed under these culture conditions [15]. Among the fractions obtained from in vitro-grown plants, the hexane fraction (FHPG) exhibited the highest yield (39.0%), followed by the aqueous fraction (30.5%), whereas the dichloromethane fraction (FDPG) accounted for only 0.5%. A similar distribution was observed in wild plants, with yields of 41.6% for the hexane fraction (FHWP), 31.0% for the aqueous fraction, and 0.5% for the dichloromethane fraction (FDWP). The predominance of the hexane fraction is consistent with the preferential extraction of lipophilic metabolites characteristic of *Acmella*, including N-alkylamides such as spilanthol, together with other nonpolar constituents such as fatty acids, terpenoids, phytosterols, and long-chain hydrocarbons [29,30].

### 2.4. Effects of Elicitation on the TLC Chemical Profile of A. radicans

**Figure 2:**
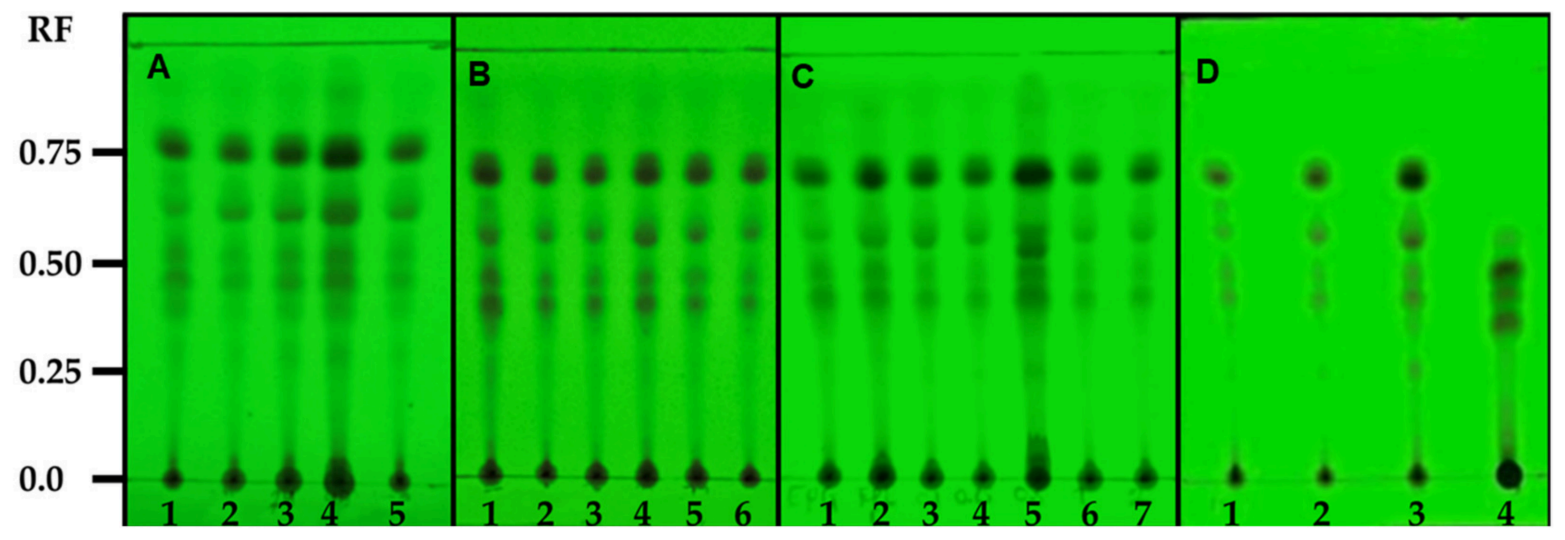
TLC profiles of extracts (**A**–**C**) and lipophilic fractions (**D**) obtained during the selection of elicitation conditions. In (**A**–**C**), lane 1 corresponds to the ethanolic extract from plants grown on gelled medium (PGE), and lane 2 to the ethanolic extract from non-elicited plants grown in liquid medium (NEE). (**A**) Plants elicited with aqueous extracts of mycelial biomass harvested at 24 h (exponential phase, lane 3), 48 h (early stationary phase, lane 4), and 72 h (late stationary phase, lane 5). (**B**) Plants exposed to the elicitor for 24 h (lane 3), 48 h (lane 4), 72 h (lane 5), and 144 h (lane 6). (**C**) Plants elicited with 0.1% (lane 3), 0.25% (lane 4), 0.5% (lane 5), 1.0% (lane 6), and 2.0% (lane 7) aqueous fungal biomass extract. (**D**) Lipophilic fractions from plants grown on gelled medium (lane 1), non-elicited plants grown in liquid medium (lane 2), plants elicited under the selected conditions (0.5% elicitor, 48 h fungal biomass, 48 h exposure) (lane 3), and wild plants (lane 4).

The resulting chromatographic profiles for mycelial age, exposure time, elicitor concentration, and the corresponding lipophilic fractions are shown in Figure 2.

At each stage, the TLC profile, based on band number and relative visual intensity, was used as a qualitative screening criterion for the sequential selection of elicitation conditions. Extract yield and antinociceptive activity of the crude extracts were not simultaneously evaluated, and the single-factor approach did not assess interactions among the variables.

#### 2.4.1. Effect of Mycelial Growth Phase

The TLC profiles were similar among treatments but differed in band intensity. At 254 nm, the band with Rf = 0.60 was more intense in non-elicited plants grown in liquid medium than in those grown on gelled medium (Figure 2A, lanes 2 and 1), consistent with the greater metabolite accumulation generally observed under liquid culture conditions [31]. Among the elicited treatments, the 48 h mycelial extract (early stationary phase) produced the greatest increase in the intensity of the bands with Rf = 0.42, 0.50, and 0.60 (Figure 2A, lane 4). This result agrees with previous studies showing that fungal elicitors prepared from cultures harvested during the late exponential or early stationary growth phases exhibit the highest elicitation efficiency because these stages are characterized by increased production of bioactive signaling molecules and elicitor-active compounds. Moreover, the effectiveness of fungal elicitors depends on factors such as fungal growth stage, elicitor composition, concentration, and induction time [25,27]. Therefore, the 48 h mycelial extract was selected for the subsequent evaluation of elicitor exposure time.

#### 2.4.2. Effect of Exposure Time

Using the selected 48 h mycelial extract, the effect of elicitor exposure time was evaluated at 24, 48, 72, and 144 h. Although the TLC profiles were similar among treatments, they differed in band intensity. At 254 nm, the 48 and 72 h treatments showed similar profiles, with higher intensities of the band at Rf = 0.50. However, the 48 h treatment (Figure 2B, lane 4) exhibited the highest intensity of the bands at Rf = 0.42 and 0.60. The band at Rf ≈ 0.50 showed chromatographic behavior similar to that previously reported for spilanthol in *Heliopsis longipes* [30]; however, this association remains tentative. Nevertheless, its presence may indicate a potentially pharmacologically relevant metabolite in *A. radicans*. The similar chromatographic profiles observed at 48 and 72 h, together with the relative band intensities, indicated no apparent additional qualitative change in the TLC profile with longer elicitor exposure, consistent with the selected elicitation window reported for plant in vitro cultures [32]. Accordingly, the 48 h exposure time was selected for the subsequent evaluation of elicitor concentration.

#### 2.4.3. Effect of Elicitor Concentration

Changes in elicitor concentration did not substantially modify the overall TLC pattern; however, differences in band intensity were observed among treatments (Figure 2C). Compared with plants grown on gelled medium (lane 1), the non-elicited liquid-medium control (lane 2) exhibited a slight increase in the intensity of the major bands. The 0.1% (lane 3) and 0.25% (lane 4) treatments produced only minor additional changes, whereas the 0.5% treatment (lane 5) exhibited the highest intensity of the bands, particularly those with Rf = 0.42, 0.50, and 0.60. Increasing the elicitor concentration to 1.0% (lane 6) reduced band intensity relative to the 0.5% treatment, whereas the 2.0% treatment (lane 7) showed a further reduction, approaching the profile of the non-elicited liquid-medium control. The 0.5% elicitor treatment produced the highest ethanol extract yield (18.8%). In contrast, both lower (0.1 and 0.25%) and higher (1.0 and 2.0%) elicitor concentrations, as well as the non-elicited control, showed lower and similar yields, ranging from 14.6 to 15.9%. This increase represents a greater amount of total ethanol-extractable material and does not necessarily indicate increased production of specific metabolites. Plants grown on gelled medium (PGE, 12.9%) and wild plants (WP, 6.1%) exhibited the lowest extract yields.

The more intense bands observed at Rf = 0.42, 0.50, and 0.60 indicate qualitative changes in the chromatographic profile following fungal elicitation. Although the band at Rf = 0.50 is close to that previously reported for spilanthol [30], TLC does not provide compound-specific identification or absolute quantification. Therefore, the increased band intensities cannot be interpreted as direct evidence of increased alkamide biosynthesis or absolute metabolite accumulation. Rather, these changes suggest that fungal elicitation modified the chemical profile of the extracts, consistent with the modulation of specialized metabolism commonly reported in response to fungal elicitors [19,33,34,35]. Similarly, fungal elicitors have been shown to enhance the expression of biosynthetic genes and the accumulation of specialized metabolites, as reported in *Panax ginseng* [33]. In contrast, excessive elicitor concentrations may impose metabolic stress that limits metabolite accumulation, highlighting the importance of optimizing elicitor dosage [27,35]. Although TLC provided a practical qualitative criterion for the sequential selection of elicitation conditions, crude extract and antinociceptive activity were not simultaneously evaluated at each step. Therefore, the selected conditions represent those producing the most pronounced changes in the TLC chemical profile rather than a comprehensive optimization of chemical yield and biological activity.

#### 2.4.4. TLC Profile of the Lipophilic Fractions

The TLC profiles of the lipophilic fractions differed among treatments (Figure 2D). Compared with plants grown on gelled medium (lane 1) and non-elicited plants grown in liquid medium (lane 2), plants elicited with 0.5% aqueous mycelial extract obtained from 48 h *R. microsporus* biomass and applied for 48 h (lane 3) exhibited increased intensities of the bands with Rf = 0.42, 0.50, and 0.60. In contrast, the lipophilic fraction from wild plants (lane 4) displayed a distinct chromatographic profile, characterized by more intense bands in the lower Rf region and a less intense band at Rf = 0.50.

Increased band intensity at Rf = 0.42, 0.50, and 0.60 indicates qualitative changes in the low-polarity metabolite profile after fungal elicitation; however, TLC does not establish increased absolute metabolite accumulation. Similar responses have been reported in elicited plant in vitro cultures, where activation of defense-related pathways stimulates the biosynthesis of specialized metabolites [32]. The band at Rf ≈ 0.50, detected in all lipophilic fractions, was tentatively associated with spilanthol based on its similarity to the Rf previously reported for *Heliopsis longipes* using the same chromatographic system [30]. The distinct chromatographic profile of wild plants indicates differences in lipophilic metabolite composition compared with in vitro-grown plants. Such differences are expected because plants growing under natural conditions are continuously exposed to multiple biotic and abiotic stimuli that modulate secondary metabolism, whereas in vitro cultures develop under controlled environmental conditions. However, TLC provides only qualitative information; therefore, the selected lipophilic fraction was subsequently characterized by GC–MS to identify its major constituents and then evaluated for its antinociceptive activity.

### 2.5. GC–MS Chemical Profiles of Lipophilic Fractions

**Figure 3:**
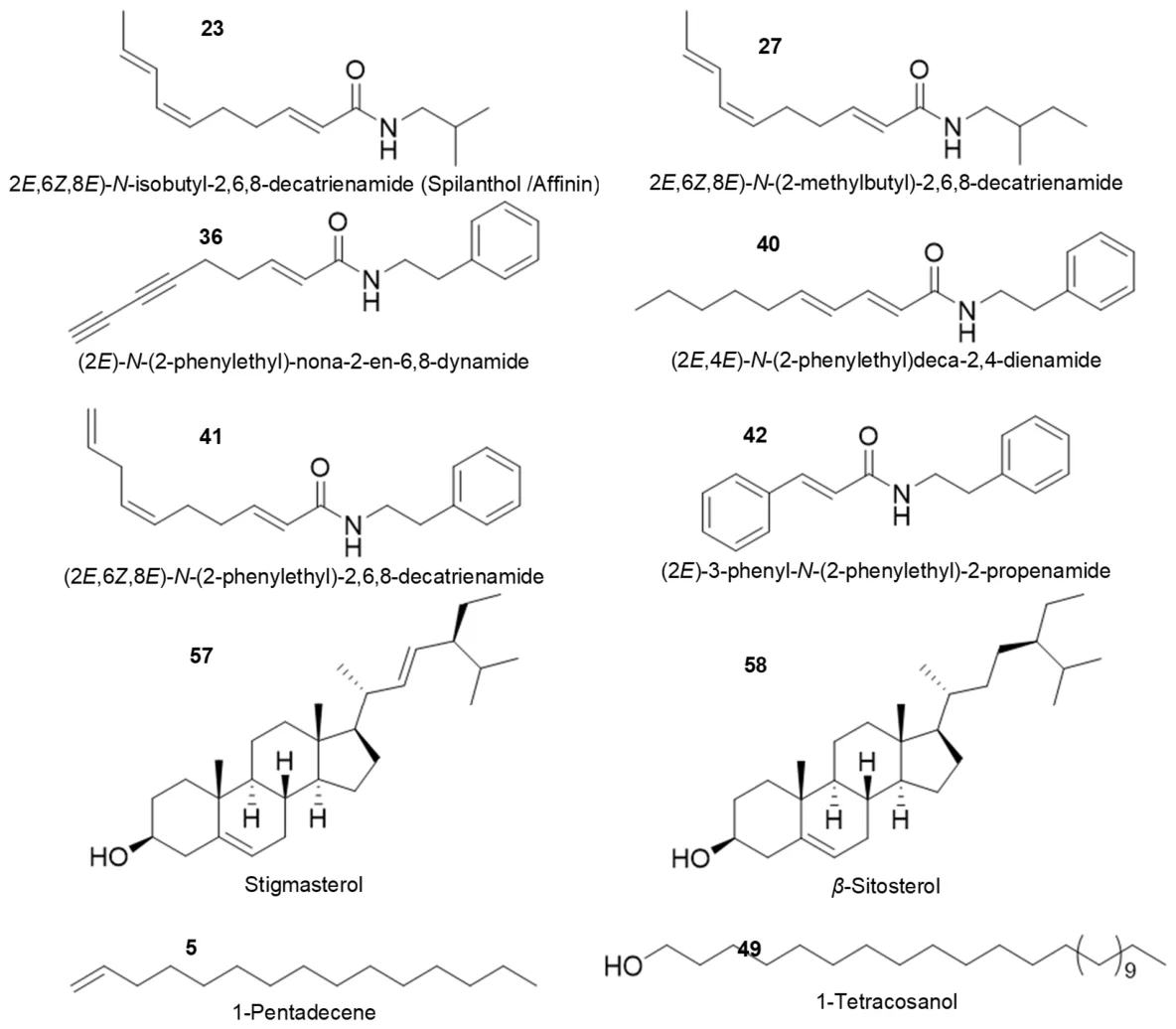
Chemical structures of the alkamides and major compounds tentatively identified by GC–MS in the lipophilic fraction of plants elicited (LFEP) under the selected elicitation conditions.

GC–MS analysis of the four lipophilic fractions (LFEP, LFNE, LFPG, and LFWP) tentatively identified 62 metabolites distributed across eight major chemical classes, including alkamides, phytosterols, diterpenes, sesquiterpenes, fatty acids and their esters, linear alkanes, monoacylglycerols, and pentacyclic triterpenes. The corresponding total ion chromatograms (TICs), provided in Figures S1–S4 (Supplementary Materials), illustrate the overall chromatographic profiles of the four fractions. The elicited fraction (LFEP) exhibited the most pronounced qualitative changes in chemical composition (Table 1, Figure 3).

Figure 3 shows the chemical structures of the alkamides, and the major compounds tentatively identified by GC–MS in the lipophilic fraction of elicited plants (LFEP) under the selected elicitation conditions.

The relative abundance of (2*E*,6*Z*,8*E*)-*N*-isobutyl-2,6,8-decatrienamide (compound **23**) was 6.42% in LFEP. In addition, several alkamides that were absent or tentatively identified only at trace levels in the other fractions were identified in LFEP, including (2*E*,6*Z*,8*E*)-*N*-(2-methylbutyl)-2,6,8-decatrienamide (compound **27**, 1.97%), (2*E*)-*N*-(2-phenylethyl)-nona-2-en-6,8-diynamide (compound **36**, 1.44%), (2*E*,4*E*)-*N*-(2-Phenylethyl)deca-2,4-dienamide (Compound **40**, 0.25%), (2*E*,6*Z*,8*E*)-*N*-(2-Phenylethyl)-2,6,8-decatrienamide (compound **41**, 0.50%), and (2E)-3-phenyl-*N*-(2-phenylethyl)-2-propenamide (compound **42**, 0.80%). The tentative identification of compound **40** in the elicited cultures is noteworthy, as, to the best of our knowledge, this compound has not previously been reported in the genus *Acmella* [12,29]. This compound has only been described in *Anacyclus pyrethrum* [36,37] and *Achillea wilhelmsii* [38], suggesting that *R. microsporus* elicitation may influence pathways associated with this unusual alkamide, although targeted studies are needed.

The proposed presence of these phenylethyl alkamides is particularly relevant because related compounds from wild *A. radicans* have been reported to exhibit potent anti-inflammatory activity through inhibition of nitric oxide and TNF-α production [12]. Moreover, several alkamides identified in *Acmella* species have been reported to exhibit antinociceptive activity, suggesting that the increased chemical diversity of alkamides in LFEP may contribute to the biological activity of this fraction [39]. In addition, LFEP contained higher relative levels of 1-Pentadecene (compound **5**, 17.75%), stigmasterol (compound **57**, 12.53%) and *β*-sitosterol (compound **58**, 6.57%). This fraction also contained metabolites detected exclusively in LFEP, including 1-tetracosanol (compound **49**, 6.97%), 2-monopalmitin (0.87%), and 2-monolinolein (0.60%) (Table 1, Figure 3). Furthermore, several metabolites showed higher relative abundance in LFEP than in the other fractions, including 2,4-di-*tert*-butylphenol (2.64% in LFEP vs. 1.12% in LFNE) and dihydroactinidiolide (0.68% in LFEP vs. 0.37% in LFPG and 0.32% in LFNE). These changes are consistent with a broader defense response induced by fungal elicitation, as these metabolites are associated with membrane remodeling, signaling, and cellular protection under stress conditions [40,41].

The non-elicited liquid-medium fraction (LFNE) and the gelled-medium fraction (LFPG) displayed lower overall chemical diversity but markedly higher relative abundances of spilanthol, reaching 11.68% and 12.77%, respectively, compared with only 0.77% in LFWP. Both fractions also exhibited increased levels of 2-tridecanone, with relative abundances of 15.32% in LFNE and 13.71% in LFPG (Table 1). These findings indicate that in vitro culture conditions markedly affect the relative abundance of this alkamide and other lipophilic metabolites, possibly as part of the physiological response to the controlled culture environment [29]. In contrast, the wild-plant fraction (LFWP) exhibited the greatest overall chemical diversity, characterized by the presence of sesquiterpenes and pentacyclic triterpene acetates, including *β*-caryophyllene (1.48%), δ-cadinene (0.52%), caryophyllene oxide (1.90%), taraxasteryl acetate (6.39%), and moretenol acetate (3.40%), all of which were absent or detected only at trace levels in the in vitro-derived fractions. Fungal elicitation markedly altered the lipophilic chemical profile of *A. radicans*. Rather than promoting further spilanthol accumulation, elicitation resulted in a distinct semi-quantitative profile characterized by structurally diverse alkamides, phytosterols, and lipid-derived metabolites (Figure 3), which may contribute to the enhanced antinociceptive activity observed in LFEP.

### 2.6. Antinociceptive Activity of Lipophilic Fractions

**Figure 4:**
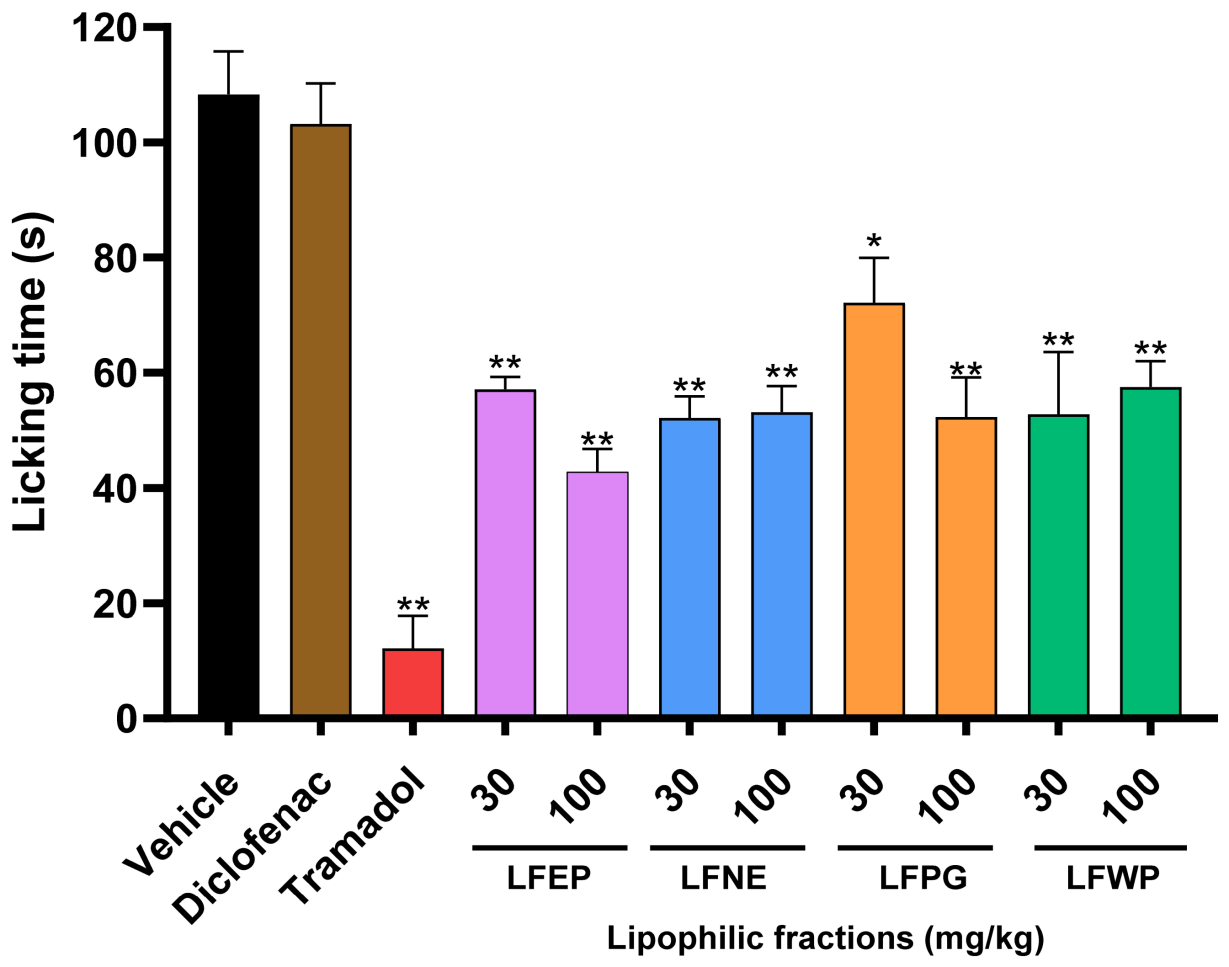
Effect of lipophilic fractions from *Acmella radicans* on the neurogenic phase (0–5 min) of the formalin-induced paw-licking test in mice. Vehicle (5% Tween), tramadol (20 mg/kg), diclofenac (20 mg/kg). Values are expressed as the mean ± SD (*n* = 6). One-way ANOVA followed by Tukey’s post hoc test. *p* < 0.05 (*), *p* < 0.001 (**) vs. vehicle. Lipophilic fraction of elicited plants (LFEP); lipophilic fraction of non-elicited plants (LFNE); lipophilic fraction of gelled-medium-grown plants (LFPG); lipophilic fraction of wild plants (LFWP).

The formalin test allowed the effects of the lipophilic fractions of *Acmella radicans* to be distinguished between the neurogenic and inflammatory phases of pain [42]. The positive controls confirmed the validity of the experimental model, as tramadol significantly reduced paw-licking behavior during both phases of the formalin test, whereas diclofenac was active only during the inflammatory phase, consistent with their known pharmacological mechanisms of action. During the neurogenic phase (Figure 4), vehicle-treated mice exhibited the longest licking time, whereas tramadol produced the greatest antinociceptive effect and diclofenac showed no significant activity. All lipophilic fractions significantly reduced licking time compared with the vehicle-treated group. LFEP exhibited a dose-dependent effect, with the 100 mg/kg dose producing the greatest inhibition among the plant fractions (60.3%), whereas tramadol produced approximately 88.7% inhibition. LFNE and LFWP significantly reduced licking time at both doses, whereas LFPG showed a weaker effect, with significant inhibition at both applied doses.

**Figure 5:**
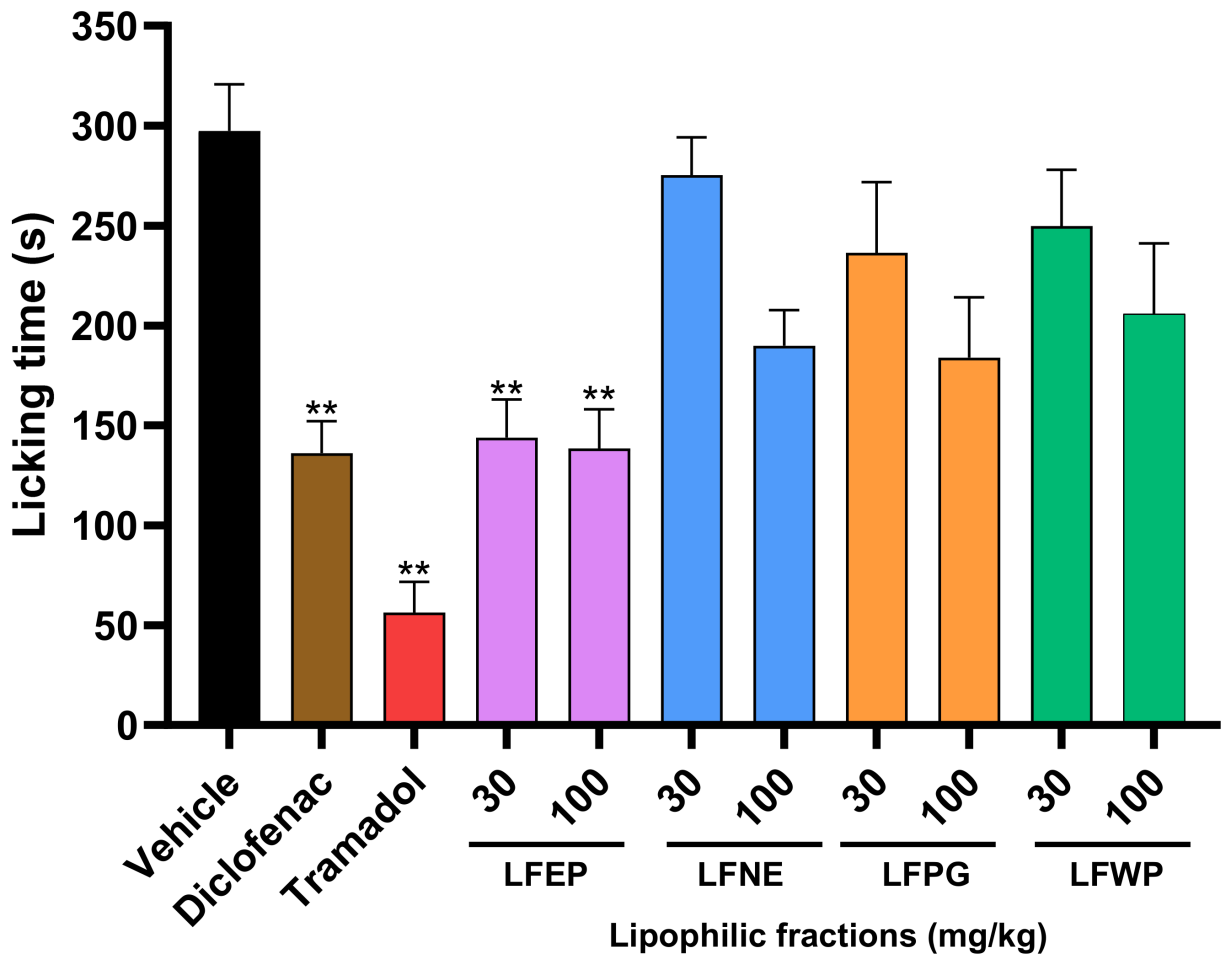
Effect of lipophilic fractions from *Acmella radicans* on the inflammatory phase (15–30 min) of the formalin-induced paw-licking test in mice. Vehicle (5% Tween), tramadol (20 mg/kg), diclofenac (20 mg/kg). Values are expressed as the mean ± SD (*n* = 6). One-way ANOVA followed by Tukey’s post hoc test. *p* < 0.001 (**) vs. vehicle. Lipophilic fraction of elicited plants (LFEP); lipophilic fraction of non-elicited plants (LFNE); lipophilic fraction of gelled-medium-grown plants (LFPG); lipophilic fraction of wild plants (LFWP).

The activity observed in the lipophilic fractions may be associated, at least in part, with spilanthol, which has previously been reported to exert antinociceptive effects through modulation of TRPV1 channels and blockade of voltage-gated sodium channels [14,39]. Given the involvement of TRPV1 in peripheral nociceptor activation during the early neurogenic phase of the formalin test [43], this mechanism may contribute to the observed activity, although further mechanistic studies are required to confirm its involvement. The effects of the lipophilic fractions during the inflammatory phase are presented in Figure 5. Vehicle-treated animals displayed the longest paw-licking time, whereas both reference drugs significantly inhibited the inflammatory response, with tramadol producing the strongest effect and diclofenac also markedly reducing licking time (*p* < 0.001). Among the plant fractions, LFEP significantly reduced the inflammatory response at both doses, with effect magnitudes approaching that observed with diclofenac (54.1% inhibition), whereas tramadol produced the strongest inhibition (81.0%). In contrast, neither LFNE, LFPG, nor LFWP showed significant antinociceptive effects at either 30 or 100 mg/kg compared with the vehicle group. Notably, although LFNE and LFWP contained relatively high proportions of spilanthol, they failed to maintain similar efficacy during the inflammatory phase, suggesting that spilanthol alone does not account for the observed response. Instead, the enhanced activity of LFEP may be associated with additional bioactive metabolites detected at higher relative abundances following fungal elicitation.

GC–MS analysis (Table 1) revealed that LFEP exhibited a qualitatively distinct chemical profile characterized by a higher abundance of *N*-phenethyl alkamides, phytosterols, and metabolites detected exclusively in this fraction. These compositional changes are pharmacologically relevant because *N*-phenethyl alkamides isolated from *A. radicans* have been reported to inhibit nitric oxide production and TNF-α release in activated macrophages, demonstrating anti-inflammatory activity beyond the modulation of nociceptive ion channels [12]. Likewise, stigmasterol and *β*-sitosterol have been associated with anti-inflammatory and antinociceptive effects, particularly during the inflammatory phase of the formalin test, through the modulation of inflammatory mediators [44]. Therefore, the enrichment of these metabolites in LFEP is consistent with its sustained activity during the second phase of the formalin assay.

In addition to these compounds, several metabolites tentatively identified exclusively or in higher abundance in LFEP may have contributed to the enhanced antinociceptive response. *β*-Caryophyllene is a selective CB2 receptor agonist that reduces inflammatory responses without producing psychoactive effects [45,46]. Similarly, alkamides from the genus *Acmella* exhibit affinity for CB1 and CB2 receptors and have been proposed to modulate the endocannabinoid system involved in pain perception [14]. Neophytadiene has also been reported to possess anti-inflammatory and neuroactive properties [47], whereas 2,4-di-*tert*-butylphenol has been identified as an HCN1 channel antagonist implicated in the regulation of neuronal excitability and pain transmission [48]. In addition, the monoacylglycerols 2-monopalmitin and 2-monolinolein may contribute to lipid-mediated signaling pathways associated with inflammatory regulation [49]. These previously reported mechanisms may provide plausible pharmacological explanations for the activity observed in LFEP.

The relative abundance of multiple compounds with previously reported anti-inflammatory and antinociceptive activities suggests that the enhanced efficacy of LFEP may result from the combined action of several bioactive constituents rather than from spilanthol alone. This interpretation is supported by the observation that fractions containing relatively high levels of spilanthol did not maintain activity during the inflammatory phase, whereas LFEP, which exhibited the most distinct chemical profile, remained active during both phases of the formalin test. These findings indicate that elicitation with *R. microsporus* altered the lipophilic chemical composition of *A. radicans* and may have contributed to the enhanced antinociceptive activity observed for LFEP. However, the contribution of individual metabolites and their underlying mechanisms of action remain to be established. Future studies should isolate, characterize, quantify, and evaluate the metabolites affected by elicitation to clarify their individual and synergistic contributions to the observed activity, as well as assess their mechanisms of action, pharmacokinetic properties, and safety. Taken together, the distinctive chemical profile of LFEP coincided with its greater antinociceptive activity in both phases of the formalin test. These findings support an association between elicitation-induced metabolic changes and enhanced antinociceptive activity; however, they do not establish a causal relationship or attribute this activity to individual metabolites.

## 3. Materials and Methods

### 3.1. Acquisition and Maintenance of the Fungal Strain

*Rhizopus microsporus* var. *oligosporus* (CDBB-H-1389) was obtained from the National Collection of Microbial Strains and Cell Cultures of the Center for Research and Advanced Studies of the National Polytechnic Institute (CINVESTAV-IPN, Mexico City, Mexico). *R. microsporus* was propagated on Petri dishes containing 20 mL of potato dextrose agar (PDA; MCD Lab, Tultitlán de Mariano Escobedo, Mexico), prepared at 39 g/L and adjusted to pH 5.6 ± 0.2. Each plate was inoculated with a 5 × 5 mm agar-free mycelial plug placed at the center. Cultures were incubated in the dark at 27 ± 2 °C and subcultured every 7 days for 3 months.

#### 3.1.1. Broth Culture and Growth Kinetics of *R. microsporus*

Agar-free mycelial fragments (5 × 5 mm) were transferred to 250 mL Erlenmeyer flasks containing 50 mL of potato dextrose broth (PDB; MCD Lab, Mexico), prepared at 24 g/L, adjusted to pH 5.6 ± 0.2, and sterilized by autoclaving at 121 °C and 18 psi for 18 min. To determine the growth kinetics of *R. microsporus*, 250 mL Erlenmeyer flasks were inoculated as described above and incubated on an AGO-6040 orbital shaker (Prendo, Puebla, Mexico) at 130 rpm and 25 ± 2 °C under a 16 h light/8 h dark photoperiod; four flasks were harvested at 6- or 12 h intervals over 120 h. Mycelial biomass was recovered by vacuum filtration through Whatman No. 1 filter paper, washed three times with distilled water, dried at 40 °C to constant weight, and weighed. The fungal growth curve was constructed based on dry biomass accumulation, and the experiment was performed three times. Based on the growth curve, mycelial biomass from the 24 h exponential phase, 48 h early stationary phase, and 72 h late stationary phase were selected for extract preparation.

#### 3.1.2. Preparation and TLC Profiling of Aqueous Mycelial Extracts

Mycelial extracts were prepared following the method of Kushwaha et al. [50]. Briefly, dried mycelial biomass was ground in a porcelain mortar and extracted with distilled water at a ratio of 1:10 (*w*/*v*). The suspension was sonicated for 10 min, autoclaved at 121 °C and 17–18 psi for 18 min, centrifuged at 1000 rpm for 10 min, and the resulting supernatant was sterilized by filtration through a sterile 0.22 µm syringe filter under aseptic conditions to obtain the aqueous mycelial extract (AME). The chemical profiles of the mycelial extracts were analyzed by thin-layer chromatography (TLC) using silica gel 60 F_254_ plates (Supelco, Sigma-Aldrich, St. Louis, MO, USA). The plates were developed using four mobile phases: 100% methanol, dichloromethane:methanol (8:2, *v*/*v*), chloroform:methanol (8:2, *v*/*v*), and *n*-butanol:acetic acid:water:methanol (4:1:3:2, *v*/*v*). The resulting TLC profiles were qualitatively compared to identify the mobile phase that provided the best separation of extract constituents. Chromatograms were visualized under UV light at 254 and 365 nm using a UVGL-25 compact UV lamp (model 95-0021-12; Analytik Jena, Upland, CA, USA).

### 3.2. Plant Material and In Vitro Micropropagation

In vitro plants of *A. radicans* were established from seeds collected from wild plants that were collected and taxonomically identified as previously described by Hurtado-Díaz et al. [12]. The seeds were surface-sterilized following the methodology previously described by Bernabé-Antonio et al. [51], and the resulting in vitro plantlets were used as the source of explants in this study. Micropropagation was performed using nodal explants cultured on full-strength Murashige and Skoog (MS) medium [52], supplemented with 3% (*w*/*v*) sucrose, plant growth regulator-free, adjusted to pH 5.8, and gelled with 2 g/L Phytagel^®^ (Sigma-Aldrich, St. Louis, MO, USA). A volume of 100 mL of medium was dispensed into 1 L glass jars and sterilized by autoclaving at 121 °C and 18 psi for 18 min. Five nodal explants were cultured per jar and incubated at 25 ± 2 °C under a 16 h light/8 h dark photoperiod for 30 days. Cultures were subsequently subcultured at regular intervals for 12 months to maintain the plant material.

### 3.3. Elicitation of In Vitro-Grown Plants Cultured in Liquid Medium

#### 3.3.1. Transfer from Gelled to Liquid Medium

Plants grown for 16 days on gelled MS medium were aseptically removed from the jars, which were rinsed with distilled water to remove residual Phytagel. Five plants were transferred to each 250 mL Erlenmeyer flask containing 50 mL of plant growth regulator-free liquid MS medium supplemented with 3% (*w*/*v*) sucrose and adjusted to pH 5.8. Cultures were incubated under orbital shaking (115 rpm) at 25 ± 2 °C with a 16 h light/8 h dark photoperiod. After 5 days, when new root emergence was observed, plants were elicited with the aqueous mycelial extract (AME).

#### 3.3.2. TLC-Guided Selection of Biotic Elicitation Conditions

The elicitation conditions were selected through a three-stage sequential approach. At each stage, only one variable was evaluated while all other experimental conditions were kept constant. The selected condition identified at each stage was used in the subsequent stage. The three stages of the selected elicitation conditions process are described below:

In the first stage, aqueous mycelial extracts obtained from the exponential, early stationary, and late stationary growth phases were applied at a fixed concentration of 2% (*w*/*v*) for 48 h [19]. In the second stage, using the extract selected from the most effective fungal growth phase, four elicitation exposure times (24, 48, 72, and 144 h) were evaluated [19]. In the third stage, while maintaining the selected extract and selected exposure time constant, five concentrations of the aqueous extract (0.10, 0.25, 0.50, 1.0, and 2.0%, *w*/*v*) were evaluated [19]. At each experimental stage, autoclave-sterilized distilled water was used as a control and applied at the same volume and under the same conditions as the treatments. In all stages, plants were harvested after the elicitation period; aerial parts and roots were separated and dried at 40 °C to constant weight. Ethanolic extracts were prepared from the dried aerial biomass of each treatment. TLC chemical profiles served as the criterion for selecting the elicitation conditions at each stage. The selected condition, based on the number and relative intensity of chromatographic bands, was carried forward to the next stage. The final selected conditions were used to produce biomass for chemical and biological analyses.

#### 3.3.3. Preparation of Ethanolic Extracts

For comparison, the aerial parts of elicited (EP) and non-elicited plants grown in liquid medium (NE), as well as 30-day-old plants grown on gelled medium (PG), were harvested, dried at 40 °C to constant weight, and ground into a fine powder. Five grams of dried biomass from both EP and NE and 200 g from PG were extracted with 96% (*v*/*v*) reagent-grade ethanol (Karal, Guanajuato, Mexico) at a biomass-to-solvent ratio of 1:10 (*w*/*v*) by maceration under continuous shaking (115 rpm) at 25 ± 2 °C for 24 h. This procedure was repeated six consecutive times to ensure exhaustive metabolite extraction. The combined extracts were vacuum-filtered and concentrated under reduced pressure using a rotary evaporator (Büchi R-3, Flawil, Switzerland) coupled to a vacuum pump (Büchi V-100). The concentrates were further dried at 40 °C to constant weight and stored at 4 °C until analysis. Extraction yield was calculated based on the initial dry biomass. The resulting extracts were designated as the ethanolic extract of elicited plants grown in liquid medium (EPE), the ethanolic extract of non-elicited plants grown in liquid medium (NEE), and the ethanolic extract of plants grown on gelled medium (PGE), yielding 900 mg, 747 mg and 26 g, respectively.

#### 3.3.4. Fractionation of Extracts and TLC Analysis of Fractions

The ethanolic extracts EPE (900 mg), NEE (747 mg), and PGE (26 g) were fractionated by liquid–liquid extraction using hexane, dichloromethane (Fermont^®^, Monterrey, Mexico), and distilled water. Briefly, each extract was suspended in 400 mL of distilled water and transferred to a separatory funnel. Hexane (150 mL) was added, and the mixture was vigorously shaken and allowed to stand for at least 15 min or until complete phase separation was achieved. The organic phase was collected, and the aqueous phase was extracted three additional times with hexane. The combined hexane fractions were concentrated under reduced pressure to obtain the corresponding hexane fractions (FHEP: 351 mg, FHNE: 291 mg, and FHPG: 10.4 g). The remaining aqueous phase was subsequently extracted three times with 150 mL of dichloromethane following the same procedure. The combined dichloromethane fractions were concentrated under reduced pressure to obtain the corresponding dichloromethane fractions (FDEP: 5.1 mg, FDNE: 3.7 mg, and FDPG: 130 mg). The remaining aqueous fractions were dried at 40 °C, and fraction yield (%) was calculated relative to the initial dry plant biomass.

The hexane and dichloromethane fractions were analyzed by TLC following the method of Molina-Torres et al. [30], using hexane:ethyl acetate (2:1, *v*/*v*) on silica gel 60 F_254_ plates (Supelco, Sigma-Aldrich, St. Louis, MO, USA). Since all fractions exhibited similar chromatographic profiles, including a spot at Rf ≈ 0.5 consistent with spilanthol [3], the corresponding hexane and dichloromethane fractions were combined to obtain the lipophilic fractions (LF) of elicited plants grown in liquid medium (LFEP), non-elicited plants grown in liquid medium (LFNE), and plants grown on gelled medium (LFPG). As a wild-plant reference, the lipophilic fraction of wild plants (LFWP) was obtained using the same liquid–liquid fractionation procedure from an ethanolic extract previously reported by Hurtado-Díaz et al. [12].

### 3.4. GC–MS Analysis of Lipophilic Fractions

The lipophilic fractions (LFEP, LFNE, LFPG, and LFWP) were dissolved in dichloromethane (5 mg/1.5 mL) and analyzed by gas chromatography–mass spectrometry (GC–MS) using an Agilent 7890B system coupled to a 5977A mass selective detector (Agilent Technologies, Santa Clara, CA, USA). Helium was used as the carrier gas at a flow rate of 1.39 mL/min on an HP-5MS UI capillary column (30 m × 0.25 mm i.d., 0.25 µm film thickness). The injector temperature was set at 250 °C, and 1 µL of each sample was injected in splitless mode. The oven temperature program was as follows: 70 °C for 1 min, increased at 10 °C/min to 180 °C, then at 5 °C/min to 280 °C, and finally at 10 °C/min to 320 °C, where it was held for 6 min. A solvent delay of 3.5 min was applied, and mass spectra were acquired in scan mode over an *m*/*z* range of 40–600. Compounds were tentatively identified by comparing their mass spectra with the Wiley10/NIST11 spectral libraries (W10N11 and W10N11_Full), and their relative abundances (%) were calculated from the corresponding peak areas in the total ion chromatogram (TIC) of each lipophilic fraction. Each lipophilic fraction was analyzed from a single GC–MS TIC profile; thus, relative abundances are semi-quantitative estimates based on peak areas.

### 3.5. In Vivo Antinociceptive Evaluation

#### 3.5.1. Animals

Male CF-1 mice (30–35 g) were obtained from the Productora Nacional de Biológicos Veterinarios (PRONABIVE; Mexico City, Mexico), accredited by the Servicio Nacional de Sanidad, Inocuidad y Calidad Agroalimentaria (SENASICA, Mexico). Male mice were selected to maintain experimental homogeneity and minimize variability associated with ovarian hormonal fluctuations. Animals were housed in the animal facility of the Centro de Investigación Biomédica del Sur (CIBIS, Xochitepec, Morelos, Mexico) under controlled environmental conditions (24 ± 2 °C, 30–40% relative humidity, 12 h light/12 h dark photoperiod, 300 lux), with free access to water and standard laboratory chow (LabDiet^®^, Quakertown, PA 18951, USA). Mice were acclimated for 7 days before experimentation. The experimental protocol was approved by the Institutional Animal Care and Use Committee at CIBIS (CICUAL-CIBIS; approval No. R-CICUAL-2025-04) and conducted in accordance with the Mexican Official Standard NOM-062-ZOO-1999 and the ethical guidelines for investigations of experimental pain in conscious animals [53,54]. Each animal was used only once and euthanized by CO_2_ inhalation at the end of the experiments.

#### 3.5.2. Treatment Preparation and Administration

For the in vivo experiments, the lipophilic fractions LFEP, LFNE, LFPG, and LFWP (Section 3.3.4) were selected. Each fraction was suspended in 5% Tween 80 at the desired concentration immediately before administration. The fractions were administered orally (p.o.) by orogastric gavage 30 min before the nociceptive assay. The negative control group received only the vehicle (5% Tween 80, 0.1 mL/10 g body weight) following the same administration protocol.

#### 3.5.3. Formalin-Induced Paw Licking Test

A total of 66 mice were allocated to eleven experimental groups (*n* = 6 per group) using a simple randomization method, with each mouse considered an independent experimental unit. No formal a priori sample-size calculation was performed. The group size was selected to obtain meaningful experimental data and minimize animal use, in accordance with the Reduction principle of the 3Rs ethical framework for animal research. No blinding was performed, and the observer recording paw-licking time was aware of treatment allocation. No animals or data points were excluded from the analysis.

Treatment groups received the lipophilic fractions LFEP, LFNE, LFPG and LFWP at doses of 30 or 100 mg/kg (p.o.) 30 min before formalin administration. The negative control group received the vehicle alone (5% Tween 80, 0.1 mL/10 g body weight). Diclofenac (AMSA^®^, 20 mg/kg, i.p.) and tramadol (AMSA^®^, 20 mg/kg, s.c.) were used as positive reference controls. Each mouse was individually placed in a cylindrical observation chamber (20 cm in diameter × 30 cm in height) equipped with rear mirrors to facilitate behavioral observation and allowed to acclimate for 30 min. Subsequently, 20 µL of 2.5% formalin was injected subcutaneously into the dorsum of the right hind paw using a 30-gauge (30G) needle. Immediately after injection, the animals were returned to the observation chamber, and the cumulative paw-licking time (s) was recorded at 5 min intervals over a 40 min observation period. Formalin injection induces a characteristic biphasic nociceptive response characterized by paw shaking and licking. Antinociceptive activity was defined as a reduction in paw-licking time compared with the vehicle-treated group [55].

### 3.6. Statistical Analysis

The growth kinetics of *R. microsporus* and the plant elicitation experiments were each conducted in three independent experiments, and quantitative data are presented as the mean ± standard deviation (SD). Antinociceptive activity data are also expressed as the mean ± SD (n = 6 animals per group). Differences among treatment groups were analyzed by one-way analysis of variance (ANOVA) followed by Tukey’s post hoc multiple comparison test using GraphPad Prism 10.5.0 (GraphPad Software, Boston, MA, USA). Differences were considered statistically significant at *p* < 0.05.

## 4. Conclusions

Fungal elicitation with *R. microsporus* modified the lipophilic chemical profile of *A. radicans* grown in vitro and increased the total ethanol extract yield. GC–MS analysis revealed marked compositional differences, including higher relative abundances of several alkamides and phytosterols and the detection of compounds absent from non-elicited fractions. These differences were accompanied by enhanced in vivo antinociceptive activity of the elicited lipophilic fraction. However, because GC–MS data were based on relative abundance, increased absolute production of individual metabolites cannot be established. Targeted quantitative analyses are therefore required to confirm changes in specific constituents. The distinctive LFEP chemical profile was associated with enhanced antinociceptive activity in both phases of the formalin test, without establishing causality or attributing the effect to individual metabolites. Overall, under the conditions evaluated, elicitation with *R. microsporus* showed potential as a biotechnological approach for modulating the chemical profile and antinociceptive activity of *A. radicans* cultivated in vitro. These findings should be interpreted considering the lack of blinding during behavioral assessment and the use of a single pain model. Future studies should isolate, characterize, quantify, and evaluate elicitation-responsive metabolites, investigate their underlying mechanisms of action, and confirm their antinociceptive effects using complementary pain models.

## Figures and Tables

**Table 1:** Tentatively identified compounds and their relative abundances (%) in the GC–MS chemical profiles of lipophilic fractions from wild, non-elicited, and elicited *Acmella radicans* plants.

No.	Compound Name	Molecular Formula	RT (min)	Match Quality (Qual)	LFEP	LFNE	LFPG	LFWP
1	*Trans*-4-Tetradecene	C_14_H_28_	8.899	97	0.10	n.d.	n.d.	n.d.
2	*β*-Caryophyllene	C_15_H_24_	9.379	99	0.80	0.47	n.d.	1.48
3	1-Pentadecyne	C_15_H_28_	9.930	98	0.47	0.98	0.68	n.d.
4	Stereoisomer of *α*-Cadinene	C_15_H_24_	10.070	98	n.d.	n.d.	n.d.	0.36
5	1-Pentadecene	C_15_H_30_	10.182	97	17.75	16.60	14.18	6.56
6	2-Tridecanone	C_13_H_26_O	10.247	97	n.d.	15.32	13.71	4.92
7	α-muurolene	C_15_H_24_	10.366	96	n.d.	n.d.	n.d.	0.24
8	2,4-Di-tert-butylphenol	C_14_H_22_O	10.423	96	2.64	1.12	n.d.	n.d.
9	Stereoisomer of *α*-Cadinene	C_15_H_24_	10.548	97	n.d.	n.d.	n.d.	0.19
10	*δ*-Cadinene	C_15_H_24_	10.635	99	n.d.	n.d.	n.d.	0.52
11	Dihydroactinidiolide	C_11_H_16_O_2_	10.750	96	0.68	0.32	0.37	n.d.
12	Spathulenol	C_15_H_24_O	11.317	99	n.d.	n.d.	n.d.	0.43
13	1-Hexadecene	C_16_H_32_	11.356	99	0.42	0.28	n.d.	n.d.
14	Caryophyllene oxide	C_15_H_24_O	11.400	94	0.24	n.d.	n.d.	1.90
15	Salvial-4(14)-en-1-one	C_15_H_22_O	11.518	96	n.d.	n.d.	n.d.	0.27
16	Carotol	C_15_H_26_O	11.547	92	n.d.	n.d.	n.d.	0.28
17	*β*-Oplopenone	C_15_H_24_O	11.686	94	n.d.	n.d.	n.d.	0.50
18	2-Pentadecanone	C_15_H_30_O	12.595	96	0.69	0.49	n.d.	n.d.
19	Ethyl myristate	C_16_H_32_O_2_	13.768	95	n.d.	n.d.	n.d.	0.18
20	Neophytadiene (I)	C_20_H_38_	14.362	99	3.61	3.83	2.64	1.24
21	Neophytadiene (II)	C_20_H_38_	14.692	95	1.00	1.10	0.76	0.49
22	Neophytadiene (III)	C_20_H_38_	14.937	90	1.68	1.84	1.00	2.50
23	(2*E*,6*Z*,8*E*)-*N*-isobutyl-2,6,8-decatrienamide	C_14_H_23_NO	15.128	90	6.42	11.68	12.77	0.77
24	Methyl palmitate	C_17_H_34_O_2_	15.550	99	n.d.	n.d.	0.67	0.90
25	*α*-Springene	C_20_H_32_	16.173	91	1.85	1.30	n.d.	n.d.
26	Ethyl palmitate	C_18_H_36_O_2_	16.535	98	0.92	0.75	2.13	5.51
27	(2*E*,6*Z*,8*E*)-*N*-(2-methylbutyl)-2,6,8-decatrienamide	C_15_H_25_NO	16.597	87	1.97	1.00	1.27	n.d.
28	Geranyllinalool	C_20_H_34_O	17.075	96	1.11	0.66	0.69	0.31
29	Nerolidol	C_15_H_26_O	17.078	94	n.d.	n.d.	0.84	n.d.
30	Methyl linoleate	C_19_H_34_O_2_	18.029	99	n.d.	n.d.	0.57	1.04
31	Methyl *α*-linolenate	C_19_H_32_O_2_	18.130	99	n.d.	n.d.	0.94	0.90
32	Phytol	C_20_H_40_O	18.304	99	3.79	2.88	4.57	4.69
33	Ethyl linoleate	C_20_H_36_O_2_	19.047	99	1.81	1.27	3.19	5.60
34	Ethyl linolenate	C_20_H_34_O_2_	19.171	99	1.40	1.04	4.24	4.88
35	Ethyl stearate	C_20_H_40_O_2_	19.558	87	n.d.	n.d.	n.d.	1.36
36	(2*E*)-*N*-(2-phenylethyl)-nona-2-en-6,8-dynamide	C_17_H_19_NO	21.152	85	1.44	n.d.	2.98	n.d.
37	Tricosane	C_19_H_40_	21.168	98	n.d.	n.d.	n.d.	3.05
38	Ethyl arachidate	C_22_H_44_O_2_	22.611	90	n.d.	n.d.	n.d.	0.53
39	Tetracosane	C_24_H_50_	22.671	97	n.d.	n.d.	n.d.	0.66
40	(2*E*,4*E*)-*N*-(2-phenylethyl)deca-2,4-dienamide	C_18_H_25_NO	22.864	83	0.25	n.d.	n.d.	n.d.
41	(2*E*,6*Z*,8*E*)-*N*-(2-phenylethyl)-2,6,8-decatrienamide	C_18_H_23_NO	22.940	94	0.50	n.d.	n.d.	n.d.
42	(2*E*)-3-phenyl-*N*-(2-phenylethyl)-2-propenamide	C_17_H_17_NO	23.646	95	0.80	0.84	1.93	0.28
43	α-Linolenic acid	C_18_H_30_O_2_	23.838	96	n.d.	1.88	n.d.	n.d.
44	1-Heptacosanol	C_27_H_56_O	24.017	95	1.45	n.d.	n.d.	n.d.
45	Cycloeicosane	C_18_H_36_	24.018	96	n.d.	1.01	0.61	n.d.
46	Pentacosane	C_25_H_52_	24.185	98	n.d.	n.d.	n.d.	2.80
47	2-Monopalmitin	C_19_H_38_O_4_	24.270	92	0.87	n.d.	n.d.	n.d.
48	2-Monolinolein	C_21_H_38_O_4_	26.851	95	0.60	n.d.	n.d.	n.d.
49	1-Tetracosanol	C_24_H_50_O	26.994	95	6.97	n.d.	n.d.	n.d.
50	Heptacosane	C_27_H_56_	27.070	98	n.d.	n.d.	n.d.	1.38
51	Ethyl tetracosanoate	C_26_H_52_O_2_	28.433	84	n.d.	n.d.	n.d.	0.57
52	Squalene	C_30_H_50_	28.859	99	1.48	0.91	6.50	1.09
53	1-Hexacosanol	C_26_H_54_O	29.784	95	1.48	0.61	0.89	n.d.
54	1-Triacontanol	C_30_H_62_O	32.414	95	1.01	n.d.	n.d.	n.d.
55	*α*-Tocopherol	C_29_H_50_O_2_	32.774	99	n.d.	1.13	1.19	n.d.
56	Campesterol	C_28_H_48_O	33.692	99	2.77	2.47	1.58	0.87
57	Stigmasterol	C_29_H_48_O	34.040	99	12.53	11.55	8.40	4.71
58	*β*-Sitosterol	C_29_H_50_O	34.578	99	6.57	6.44	4.94	5.18
59	*β*-Amyrin	C_30_H_50_O	34.857	99	n.d.	n.d.	n.d.	1.93
60	12-Oleanen-3-yl acetate	C_32_H_52_O_2_	35.746	98	n.d.	n.d.	n.d.	3.78
61	Moretenol acetate	C_32_H_52_O_2_	36.816	89	n.d.	n.d.	n.d.	3.40
62	Taraxasteryl acetate	C_32_H_52_O_2_	36.929	95	n.d.	n.d.	n.d.	6.39

LFEP, lipophilic fraction of elicited plants; LFNE, lipophilic fraction of non-elicited plants; LFPG, lipophilic fraction of gelled-medium-grown plants; and LFWP, lipophilic fraction of wild plants. Qual: spectral library match quality obtained by comparison with the Wiley10/NIST11 mass spectral libraries.

## Data Availability

The raw data supporting the conclusions of this article will be made available by the authors on request.

## Supplementary Materials

The following supporting information can be downloaded at: https://www.mdpi.com/article/10.3390/molecules31162906/s1, Figure S1: Total ion chromatogram (TIC) obtained by GC–MS analysis of the lipophilic fraction of *Acmella radicans* plants elicited under the selected conditions (0.5% *R. microsporus* mycelial extract, 48-h fungal biomass, and 48-h exposure) (LFEP); Figure S2: Total ion chromatogram (TIC) obtained by GC–MS analysis of the lipophilic fraction of non-elicited *Acmella radicans* plants grown in liquid medium (LFNE); Figure S3: Total ion chromatogram (TIC) obtained by GC–MS analysis of the lipophilic fraction of *Acmella radicans* plants grown on gelled medium (LFPG); Figure S4: Total ion chromatogram (TIC) obtained by GC–MS analysis of the lipophilic fraction of wild *Acmella radicans* plants (LFWP).
