# Supplementary material for: Enhanced In Vivo Antinociceptive Activity and GC–MS Chemical Profiling of In Vitro-Grown Acmella radicans Plants Elicited with Rhizopus microsporus
Source: Molecules. 2026 Aug 20;31(16):2906. doi: 10.3390/molecules31162906 (PMC13515771; doi:10.3390/molecules31162906)
Supplement: Supplementary file 1 [file molecules-31-02906-s001.zip › molecules-4483472-supplementary.pdf]

Supplementary material

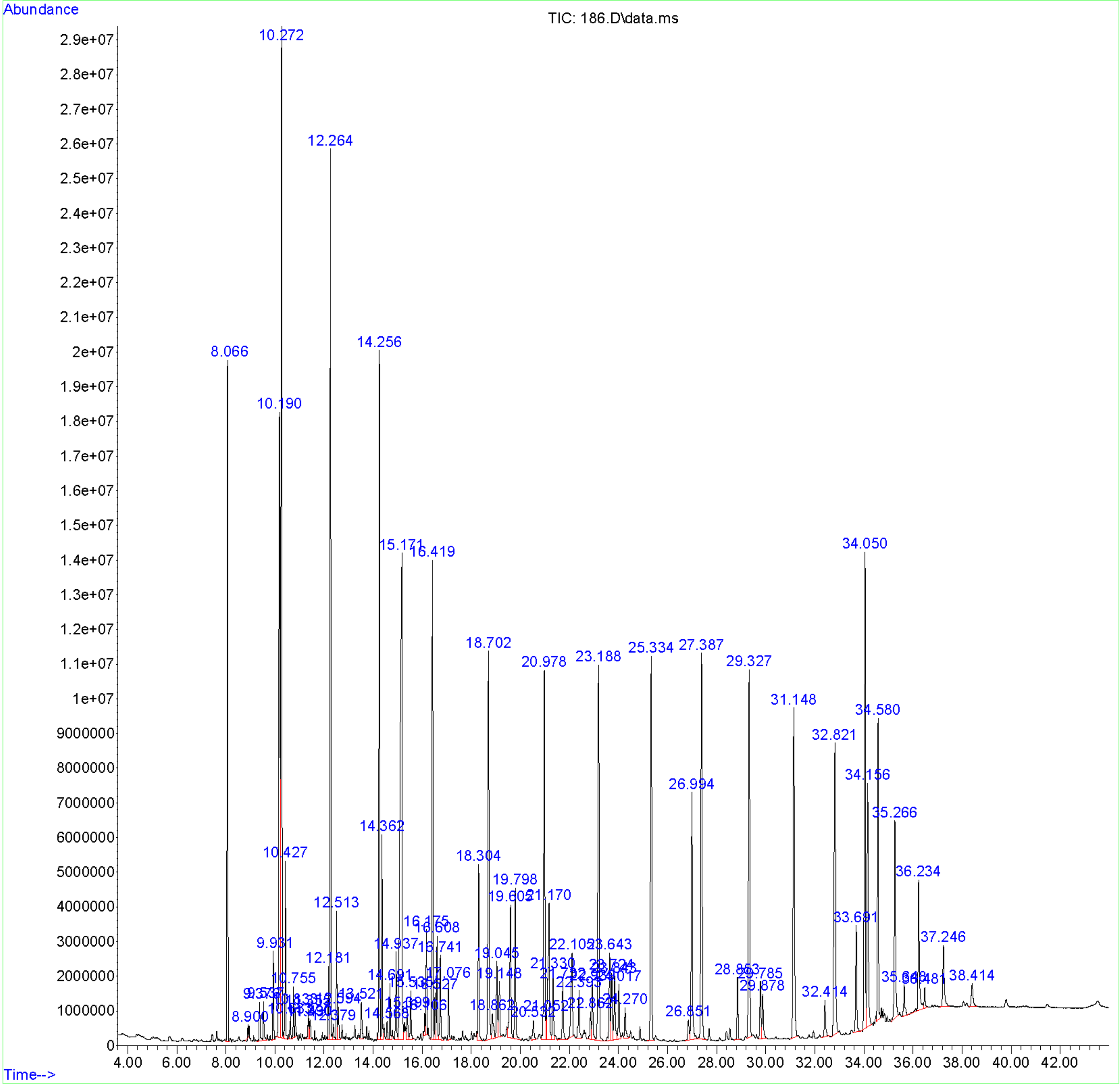

Figure S1. Total ion chromatogram (TIC) obtained by GC–MS analysis of the lipophilic fraction of *Acmella radicans* plants elicited under the selected conditions (0.5% *R. microsporus* mycelial extract, 48-h fungal biomass, and 48-h exposure) (LFEP).

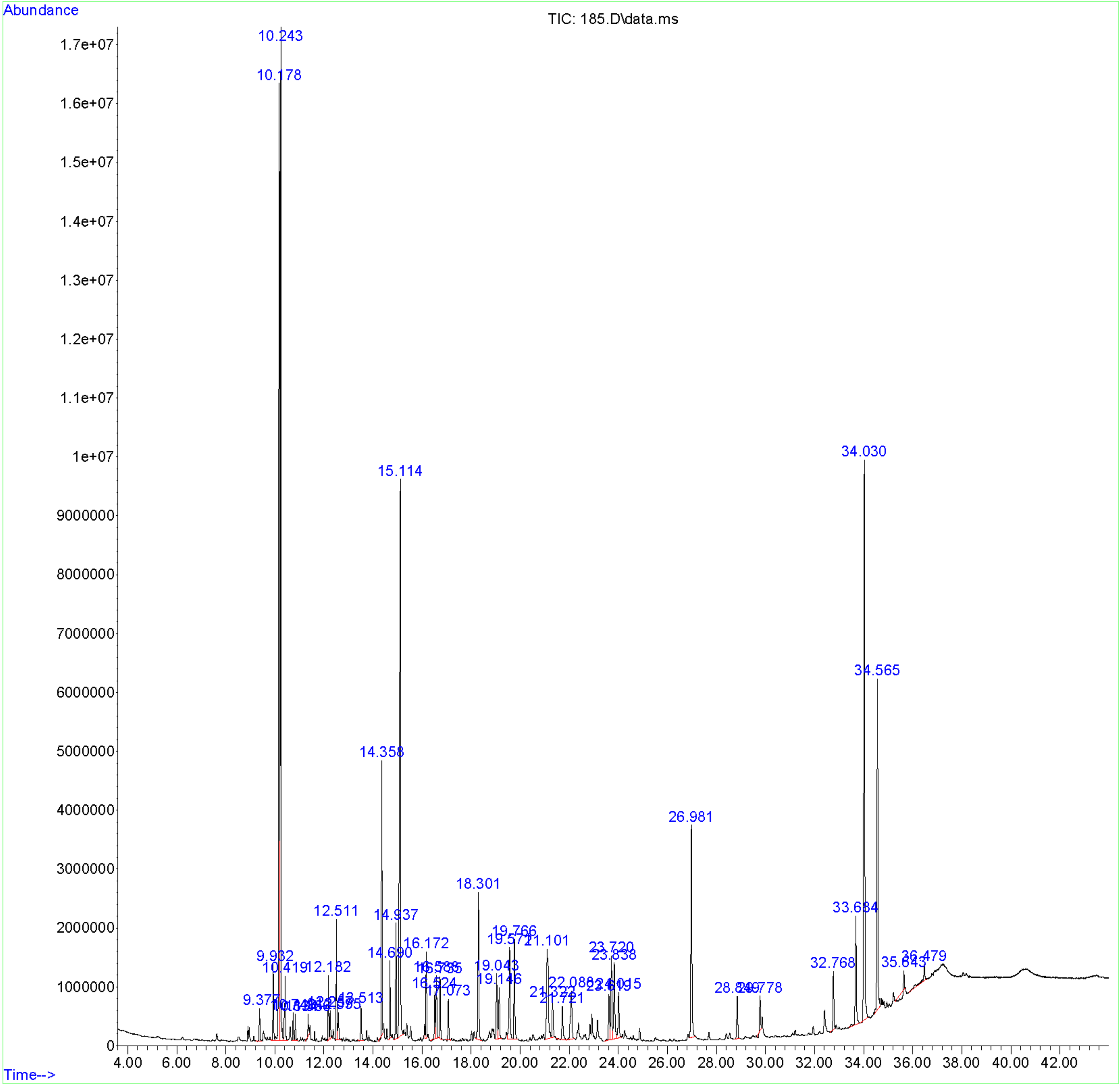

Figure S2. Total ion chromatogram (TIC) obtained by GC–MS analysis of the lipophilic fraction of non-elicited *Acmella radicans* plants grown in liquid medium (LFNE).

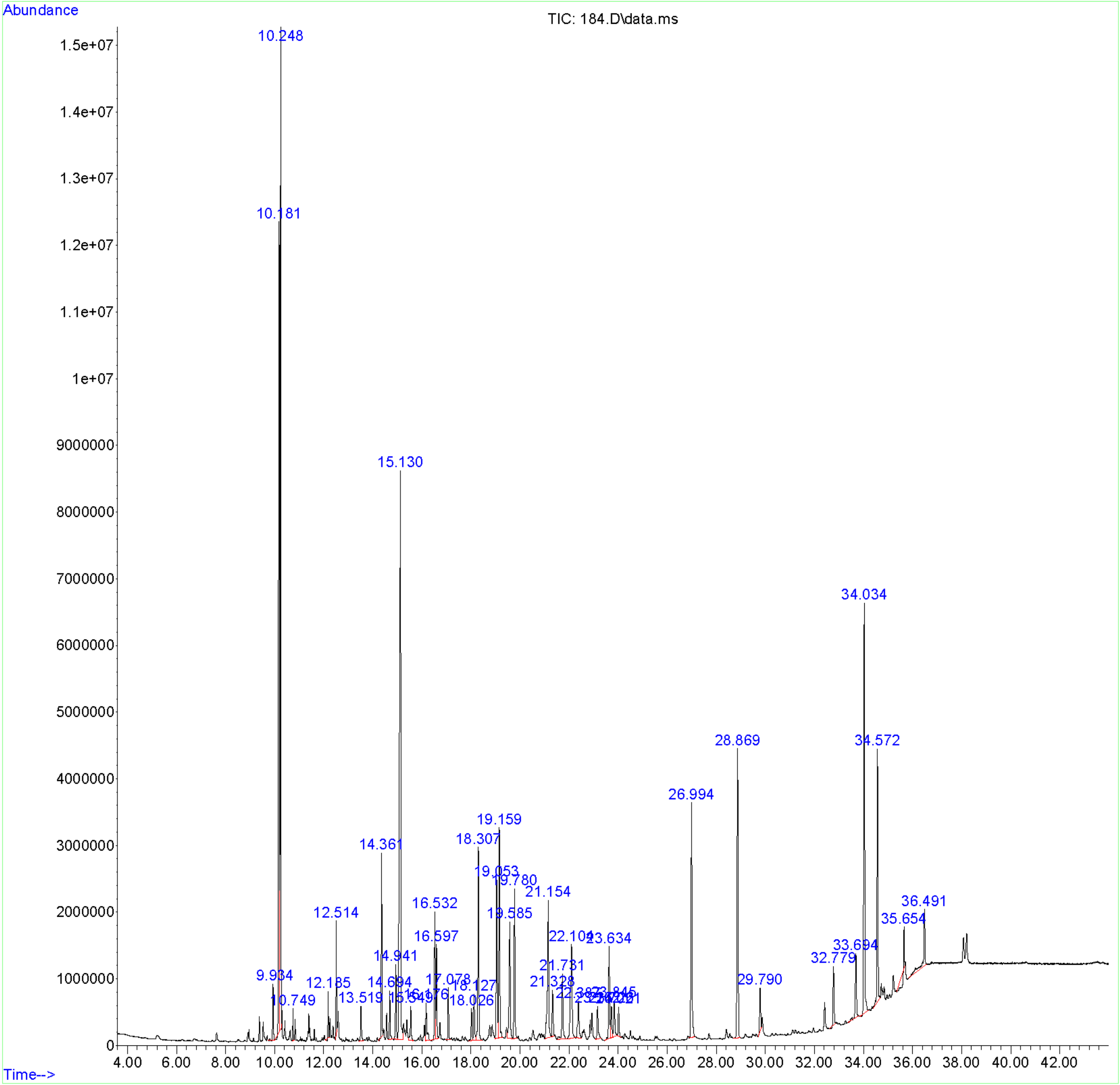

Figure S3. Total ion chromatogram (TIC) obtained by GC–MS analysis of the lipophilic fraction of *Acmella radicans* plants grown on gelled medium (LFPG).

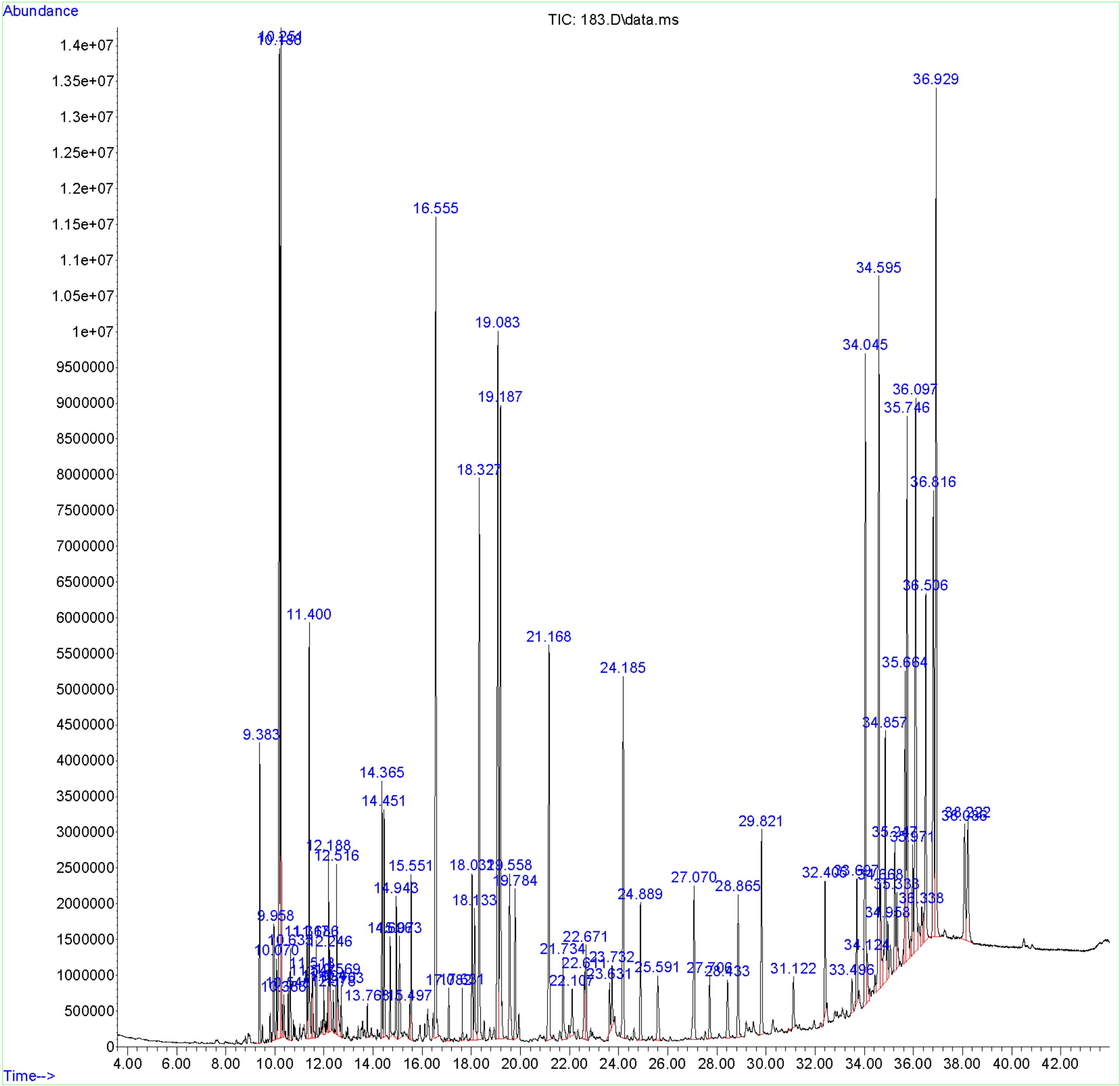

Figure S4. Total ion chromatogram (TIC) obtained by GC–MS analysis of the lipophilic fraction of wild *Acmella radicans* plants (LFWP).
